# SLAM Back-End Optimization Algorithm Based on Vision Fusion IPS

**DOI:** 10.3390/s22239362

**Published:** 2022-12-01

**Authors:** Yu Xia, Jingdi Cheng, Xuhang Cai, Shanjun Zhang, Junwu Zhu, Liucun Zhu

**Affiliations:** 1 School of Information Engineering, Yangzhou University, Yangzhou 225127, China; 2 Advanced Science and Technology Research Institute, Beibu Gulf University, Qinzhou 535011, China; 3 Research Institute for Integrated Science, Kanagawa University, Kanagawa 259-1293, Japan

**Keywords:** SLAM, Indoor Positioning System, back-end optimization, positional trajectory

## Abstract

SLAM (Simultaneous Localization and Mapping) is mainly composed of five parts: sensor data reading, front-end visual odometry, back-end optimization, loopback detection, and map building. And when visual SLAM is estimated by visual odometry only, cumulative drift will inevitably occur. Loopback detection is used in classical visual SLAM, and if loopback is not detected during operation, it is not possible to correct the positional trajectory using loopback. Therefore, to address the cumulative drift problem of visual SLAM, this paper adds Indoor Positioning System (IPS) to the back-end optimization of visual SLAM, and uses the two-label orientation method to estimate the heading angle of the mobile robot as the pose information, and outputs the pose information with position and heading angle. It is also added to the optimization as an absolute constraint. Global constraints are provided for the optimization of the positional trajectory. We conducted experiments on the AUTOLABOR mobile robot, and the experimental results show that the localization accuracy of the SLAM back-end optimization algorithm with fused IPS can be maintained between 0.02 m and 0.03 m, which meets the requirements of indoor localization, and there is no cumulative drift problem when there is no loopback detection, which solves the problem of cumulative drift of the visual SLAM system to some extent.

## 1. Introduction

**Figure 1:**
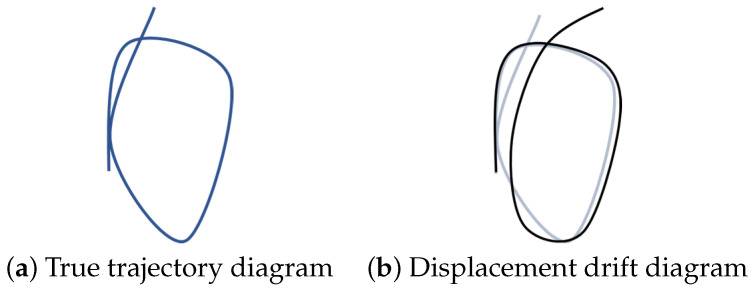
Schematic diagram of cumulative drift in VSLAM.

Simultaneous Localization and Mapping (SLAM) refers to the indoor environment in which the Global Positioning System (GPS) or BeiDou Navigation Satellite System (BDS) module does not receive satellite signals and cannot obtain location-related information such as latitude and longitude, the robot uses the features in the environment to estimate its pose; and extracts the relevant location and other information of all objects in the unknown environment, i.e., to describe the environment. The robot not only needs to localize itself using the created map but also needs to update the environment map based on the current pose information. In Visual SLAM (VSLAM), the visual odometry (VO) will accumulate errors over a long period resulting in unreliable estimated positional trajectory results. This will lead to the inability of the whole VSLAM system to obtain globally consistent positional trajectory information, which will have a large impact on the results of both localization and map building. The accumulated drift phenomenon in visual SLAM is shown in Figure 1.

For the cumulative drift problem, the classical framework of visual SLAM uses loopback detection processing, which determines whether the robot returns to the previously passed position by detecting the similarity between two frames, i.e., whether there is a loopback in the travel trajectory. If the mobile robot can detect the loopback while performing the task, and the constraints established by the loopback are added to the back-end optimization can ensure that the acquired positional trajectory has global consistency and the estimated trajectory results are closer to the real trajectory. However, the drawback of loopback detection is also very obvious, if the mobile robot does not detect loopback during operation (e.g., moving in a straight corridor), it cannot use loopback to correct the positional trajectory, and the cumulative drift problem of the visual SLAM system remains unsolved.

In order to solve the cumulative drift problem of the visual SLAM system in the loopback-free task, borrowing the idea of using GNSS in the outdoor environment, this paper adds Indoor Positioning System (IPS) to the back-end optimization of VSLAM to provide global constraints for the optimization of the positional trajectory and proposes a back-end optimization framework based on the positional map. Firstly, the VO information is obtained from the front-end VO calculation method, and then the bit-pose information obtained by IPS adaptive outlier suppression algorithm is optimized by the IPS adaptive outlier suppression algorithm. Finally, the constructed residual terms are constructed from the input data and added to the pose-map optimization through the pose-map optimization module to calculate the transformation relationship from VO to its coordinate system. We conducted experiments on the AUTOLABOR mobile robot, and the results proved that the problem of cumulative drift of the VSLAM system can be solved to a certain extent.

## 2. Related Work

The mainstream sensor types used for SLAM are laser sensors and vision sensors. Laser SLAM is relatively more accurate, but LIDAR is more expensive and has a limited detection range, in addition to the lack of semantic information and the difficulty of loopback detection. In environments with poor environmental scene diversity, such as structured corridors or tunnels with consistent structure and other structured scenes, degradation problems will occur, and at this time, laser SLAM is more prone to degradation problems compared to VSLAM. In contrast, VSLAM has the advantages of low cost, small size, low power consumption, and rich information perception, and is more suitable for indoor environment.

One of the more common problems in VSLAM systems is the cumulative drift problem. Each calculation and optimization of the visual front-end of SLAM generates a small error, and when the camera moves continuously for a long time, this small error will accumulate and eventually cause the estimated trajectory to deviate from the real motion trajectory. Feature-based SLAM associates feature extraction (e.g., SURF [1], SIFT [2] and ORB [3]) and matching with data, and then performs pose estimation by minimizing the feature point reprojection error. Direct VSLAM treats data association and pose estimation as an overall optimization problem and solves the problem by minimizing the photometric error. The reliance on accurate data correlation can lead to tracking failures for low textures, lighting variations, and occluded areas. In addition, geometric methods have the inherent disadvantage that camera motion can only be estimated to an unknown scale, which can also lead to scale drift over time.

A.J. Davison [4] proposed the first monocular SLAM system, MonoSLAM, in which the back-end optimization part used is based on the Extended Kalman Filter (EKF) optimization method. Subsequently, Eade et al. [5] improved the SLAM back-end optimization algorithm proposed by A.J. Davison based on MonoSLAM using Particle Filter (PF) algorithm and proposed a Fast-SLAM based SLAM back-end optimization scheme. The VSLAM system alleviates this problem by combining geometric VO with an optimized backend to continuously adjust the position of landmarks and the pose of the camera, called graph optimization technique. Graph-based SLAM has been successful in many cases in established systems, such as feature-based PTAM [6] and ORB-SLAM [7], as well as direct LSD-SLAM [8] and DSO [9]. The graph-based SLAM [10] maintains a global graph with nodes representing camera poses or landmarks and edges representing sensor measurements that constrain the connected poses. In the back-end optimization process, the traditional filter-based approach is replaced with a graph optimization approach, which provides an important pavement for later graph optimization schemes.

In SLAM, with the help of loop closure, i.e., graph optimization with correctly established loop closure constraints, SLAM is able to significantly reduce global trajectory drift. Most of the existing LIDAR-based closed-loop detection is achieved by encoding the point cloud as global or local descriptors and then matching the descriptors. They usually use low-level features, such as coordinates [11,12,13,14,15], normal [16], reflection intensity [17,18,19,20], etc. In recent years, with the development of deep learning, many LiDAR-based methods for target detection [21] and semantic segmentation [22,23] have been proposed, making it possible to obtain semantic information from point clouds. However, still only a few LIDAR-based works attempt to use semantic information [24,25].

In the field of VSLAM, closed-loop detection based on local features [1,3] and Bag-of-Words (BoW) [26] are well established and have been widely used [27,28]. Unlike images that contain rich texture features, point clouds are almost purely geometric information, which makes point cloud-based closed-loop detection challenging. Therefore, there is no effective method to integrate into LiDAR SLAM systems. The ranging and mapping algorithms represented by LOAM [29] achieved very high accuracy on the KITTI [30] dataset.

The most currently used solution for the cumulative drift problem in VSLAM is still loopback detection. In an outdoor environment, Autoware [31], an autonomous driving framework in Shinpei Kato lab, uses a Global Navigation Satellite System (GNSS) to provide global constraints for 3D laser SLAM localization, which not only solves the cumulative drift problem of the positional trajectory but also ensures that the LiDAR performs point cloud map matching localization with better initial values. In current VSLAM systems in indoor environments, Yang B. [32] proposed a novel multi-class and motion attribute concurrent VSLAM (MCV-SLAM) algorithm to ensure that VSLAM works properly in real-time in dynamic indoor environments with moving objects. Hu H. [33] proposed a multi-map-based SLAM system with a four-thread structure using multiple small maps for tracking to reduce the error accumulation of VSLAM and introduced an algorithm to predict camera pose to improve the robustness of tracking. Tseng P.-Y. [34] proposed an economical indoor localization system based on visual simultaneous localization and mapping (vSLAM) with low setup cost and an integrated approach for precise positioning using vSLAM to solve the matching and accuracy. Raza A. [35] explored various indoor localization techniques which compared and contrasted various approaches and classified them according to a set of suggested criteria to evaluate a commercially deployable indoor localization solution. However, there is no more research and better solutions for solving the SLAM cumulative drift problem in loopback-free tasks in indoor environments. In this paper, the proposed solution of adding IPS to the back-end optimization of VSLAM solves the cumulative drift problem of indoor VSLAM systems in loopback-free tasks to some extent.

## 3. A Back-End Optimization Framework Based on Positional Maps

**Figure 2:**
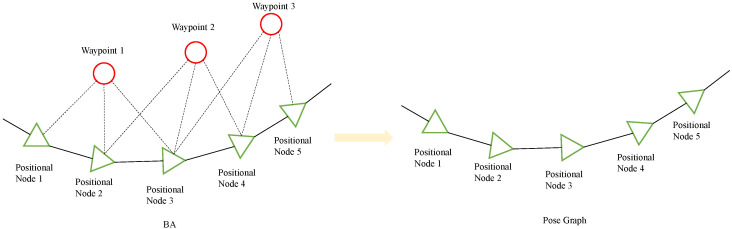
Schematic diagram of BA optimization compared with pose-map optimization.

The front-end motion position estimation requires feature points (waypoint) to be involved in optimally estimating the robot motion. In fact, after several observations, the converged feature points (waypoint) have little positional transformation, while the outlier points are eliminated. It is more redundant to optimize again for the points that have already converged. Therefore, when the motion estimation smoothes out, the pose-map optimization will no longer optimize the positions of feature points (waypoint), but only the connections between all robot poses. In this way, a large number of feature point optimization calculations can be saved while only the trajectories of key frames are retained. Figure 2 shows the comparison between Bundle Adjustment (BA) optimization and pose map optimization.

The traditional bitmap-based optimization algorithm contains only the relative bitmap constraints of two adjacent frames, and optimizes the bitmap data within a certain time range through the bitmap. However, because the back-end optimization only considers the constraints of adjacent frames, it is not effective for cumulative error elimination. In order to solve the cumulative error problem of SLAM system and construct globally consistent trajectories and maps, the global positional information of IPS is considered to be added to the positional map, so that the positional map has global positional constraints.

**Figure 3:**
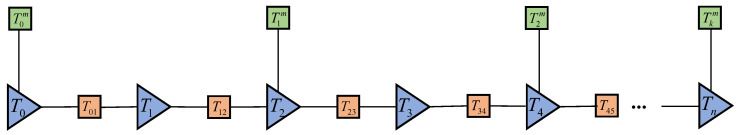
Optimization of pose map with IPS constraints.

As shown in Figure 3, $T_{1},\cdots ,T_{n}$ nodes represent the robot poses, and the motion between two poses is obtained by the VO. Assuming that a motion $T_{ij}$ between $T_{i}$ and $T_{j}$ has been estimated, the relative bit pose between frame *i* and frame *j* can be expressed on the Lie group SE(3) as $T_{ij}=T_{i}^{-1}T_{j}$. To make the relative bit pose between $\left\{x_{0},x_{1},x_{2},\cdots ,x_{n}\right\}$ of these moments of the poses $\left\{T_{0},T_{1},T_{2},\cdots ,T_{n}\right\}$ optimizes two aspects of the observations that can be used, firstly, the poses of the moving robot at each moment obtained by IPS, which have no cumulative error but are released at a low frequency of about 5–8 Hz, and is represented by $\left\{T_{0}^{m},T_{1}^{m},T_{2}^{m},\cdots ,T_{k}^{m}\right\}$. Next is the VO pose obtained from the front-end pose estimation, this observation has a high release frequency of about 20–25 Hz and the high accuracy in short time is given by by $\left\{T_{01},T_{12},T_{23},\cdots ,T_{(n-1)n}\right\}$. Based on the above characteristics, the fusion strategy adopted in this paper is to use IPS as the absolute constraint of the positional map, and use VO to establish relative constraints between two frames of IPS poses, and since the VO frequency and the release frequency of IPS are different, it is necessary to perform time matching on them before optimization.

In order to facilitate the solution of the Gaussian Newton method, it is necessary to derive the Jacobi matrix of the error terms of the IPS observations with respect to the bit pose. Since the observation in the lattice diagram is a unitary edge connecting only one lattice state quantity, the observation of this state quantity is given directly. IPS constraint corresponds to the residual that is the difference between the observation and the state quantity as shown in Equation (1). Adding a perturbation to the residuals in Equation (1) yields Equation (2). The simplification of Equation (2) using the concomitant property Equation (3) and the BCH formula yields Equation (4), where the residuals are Jacobi with respect to $T_{i}$ as Equation (5). $J_{r}^{-1}\left(e_{i}\right)$ has the form $J_{r}^{-1}\left(e_{ij}\right)\approx I+\frac{1}{2}\left[\begin{array}{cc} \phi_{e}^{\wedge } & \rho_{e}^{\wedge }\\ 0 & \phi_{c}^{\wedge } \end{array}\right]$.

(1) $$e_{i}={ln\left(Z_{i}^{-1}T_{i}\right)}^{\vee }={ln\left(exp\left({\left(-\xi_{zi}\right)}^{\wedge }\right)exp\left(\xi_{i}^{\wedge }\right)\right)}^{\vee }.$$

(2) $${\hat{e}}_{i}={ln\left(Z_{i}^{-1}exp\left(\delta \xi_{i}^{\wedge }\right)T_{i}\right)}^{\vee }.$$

(3) $$\left\{\begin{array}{c} Texp\left(\xi^{\wedge }\right)T^{-1}=exp\left({\left(Ad\left(T\right)\xi \right)}^{\wedge }\right)\\ Ad\left(T\right)=\left[\begin{array}{cc} R & t^{\wedge }R\\ 0 & R \end{array}\right] \end{array}\right..$$

(4) $${\hat{e}}_{i}\approx e_{i}+\frac{\partial e_{i}}{\partial \delta \xi_{i}}\delta \xi_{i}.$$

(5) $$\frac{\partial e_{i}}{\partial \delta \xi_{i}}=J_{r}^{-1}\left(e_{i}\right)Ad\left(T_{i}^{-1}\right).$$

To construct the least squares problem with IPS optimization added, the observed value of IPS at k moments is denoted as $T_{k}^{M}$, then the IPS error term at *k* moments can be denoted as $e_{k}^{m}=T_{k}^{-1}T_{k}^{m}$.where $T_{k}$ is the attitude estimate at moment *k*. The error term of the front-end VO pose estimation is expressed as $e_{ij}^{vo}={\left(T_{ij}^{vo}\right)}^{-1}T_{i}^{-1}T_{j}$, $T_{ij}^{vo}$ is the visually observed pose transformation from moment *i* to moment *j*.

All the positional vertices and positional edges can form a graph optimization, which essentially constitutes a least squares problem, where the edges are constraints on the positional observations, and the optimization variables are the poses of the individual vertices. Writing all the error terms as an overall objective function can be expressed as Equation (6):

(6) $$min\frac{1}{2}\sum_{i,j\in \varepsilon }e_{ij}^{T}\sum_{ij}^{-1}e_{ij},$$

where ε denotes the set of all optimized edges, $\sum_{ij}^{-1}$ is the information matrix, inverse matrix of the covariance matrix, indicating the magnitude of the weights, $e_{ij}$ is the error term.

The back-end positional optimization can be achieved by solving Equation (7). The entire optimization problem can be defined as follows:

(7) $$min\frac{1}{2}\left(\sum_{i,j\in \varepsilon }{\left(e_{ij}^{vo}\right)}^{T}\sum_{ij}^{-1}e_{ij}^{vo}+\sum_{k\in \varepsilon }{\left(e_{k}^{m}\right)}^{T}\sum_{k}^{-1}e_{k}^{m}\right).$$

After optimizing the construction into a nonlinear least squares problem using the positional map, the global poses are optimized by invoking the methods in the Ceres library for solving the optimal global poses.

## 4. Back-End Optimization Algorithm for Visual Fusion IPS

### 4.1. Overall Algorithm Flow

The whole fusion algorithm is divided into three main parts. The first part is the data reading module, which obtains the VO information from the front-end VO calculation method. The bit attitude information is read from the IPS, where the attitude information is the heading angle, which is obtained by the double label orientation method. After getting the VO data and IPS data use the data time matching function that comes with the ROS system to align their data timestamps.

The second part is the IPS outlier removal module. The outlier suppression algorithm proposed in this paper is used for the position and attitude information obtained by IPS. The algorithm first needs to calculate the motion of adjacent frames of VO and IPS in the same time, then calculate the motion error between them, and finally determine whether the error is less than the threshold value based on whether the IPS data at that time is added to the optimization.

**Figure 4:**
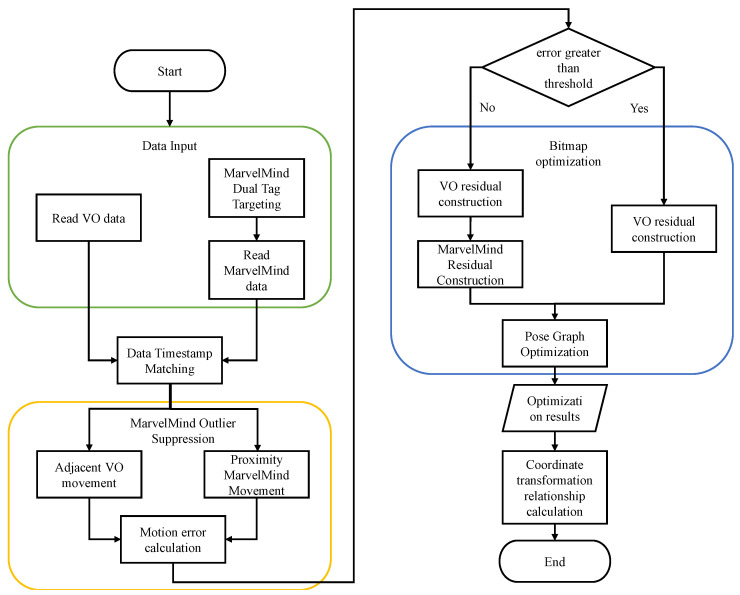
Flow of IPS and VO fusion algorithm.

The third part is the pose map optimization module, which is based on Ceres library. First, according to the input data, choose to construct VO residual and IPS residual at the same time or only construct VO residual. Then, add the constructed residual to the pose map for optimization. After the optimization, calculate the transformation relationship between VO and IPS coordinate system according to the optimization results, and then carry out the next round of optimization. The overall flow chart is shown in Figure 4.

### 4.2. IPS Adaptive Outlier Suppression Algorithm

In the static state indoors, the accuracy of the position information obtained by IPS can be maintained at about 2 cm. However, in the actual environment, it is found that the IPS signal may be blocked by objects and environmental noise at some moments in the mobile state, which may lead to a decrease in the accuracy of the received position at a certain moment, and then the fusion of the acquired outliers with the VO will definitely affect the final result. In order to solve this problem, in this paper, we first perform the outlier rejection process on the acquired IPS information before performing the positional fusion. Inspired by the adaptive GNSS outlier rejection algorithm proposed in Das [36], this section proposes an outlier suppression algorithm for the IPS positioning module to pre-process the IPS data before fusion and eliminate the influence of outliers on the system.

The front-end pose estimation only contains the pose constraint between two adjacent frames, and although the VO results of long-time operation are not credible, the pose information obtained by it has higher accuracy in a short period of time. While the global positional information obtained by IPS is more accurate, the positional information obtained in a short period of time is susceptible to jumps due to external noise interference, and there is a complementary relationship between these two frames. Based on the above characteristics, the VO acquired by the front-end is used as the observation value to reject the abnormal values of the IPS positioning information. When a new IPS measurement is acquired, the relative change between the current measurement and the previous moment measurement is calculated, and the error between the VO relative to the measurement over the same time span is also calculated. If the error of heading (position) change is less than 1.5${}^{\circ }$ ($H_{head}$) and the displacement change is less than 0.02 m ($H_{position}$), the current IPS position information is added to the optimization, otherwise the information is discarded. The specific algorithm flow is shown in Algorithm 1.
**Algorithm 1:** IPS adaptive outlier suppression algorithm** Input:** Threshold $H_{head}$; $H_{position}$; Current IPS Positions $T_{Mnow}$; Current VO position $T_{VOnow}$
 Step 1: Calculate the IPS positional change $\Delta T_{M}=T_{Mlast}^{-1}T_{Mnow}$, Calculate the VO
positional change $\Delta T_{VO}=T_{VOlast}^{-1}T_{Onow}$.
 Step 2: Update the previous moment’s pose data with the current pose data
$T_{Mlast}$, $T_{VOlast}$.
 Step 3: IPS pose change $\Delta R_{M}$ and displacement change $\Delta t_{M}$ are extracted from the
change matrix $\Delta T_{M}$.
 Step 4: VO pose change $\Delta R_{VO}$ and displacement change $\Delta t_{VO}$ are extracted from
the change matrix $\Delta T_{VO}$.
 Step 5: Substitute $\Delta R_{M}$, $\Delta R_{VO}$, $\Delta t_{M}$, $\Delta t_{VO}$ into the equation $S=\sqrt{\sum_{i=0}^{m}\sum_{j=0}^{n}{\left(A_{ij}-B_{ij}\right)}^{2}}$ respectively The error of change of heading angle *e_R_* and the error of change of displacement *e_t_*.
 Step 6: **if**
$e_{t}\leq H_{position}e_{R}\leq H_{head}$.
 Step 7: Add the current IPS pose $T_{Mnow}$ to the pose map.
 Step 8; **else**.
 Step 9: The current IPS pose does not join $T_{Mnow}$.


## 5. Experimental Results and Analysis

### 5.1. Experimental Hardware Environment

**Figure 5:**
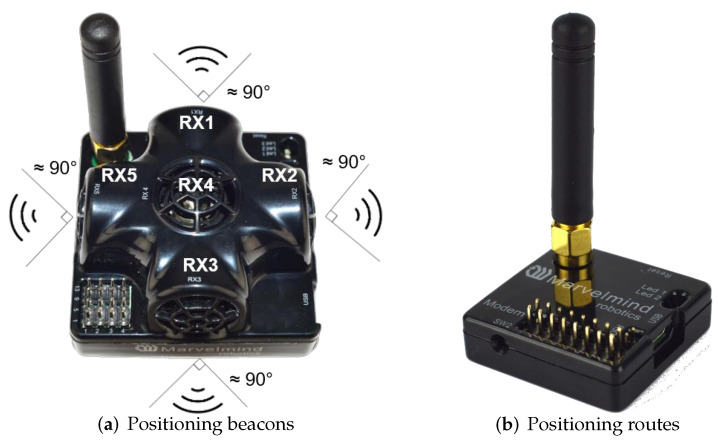
Composition of MarvelMind positioning module.

In this paper, we use the MarvelMind indoor positioning module [37] as the fused IPS sensor. MarvelMind consists of three components: fixed beacons, mobile beacons, and localization routing. The fixed beacons are placed within the working area of the mobile robot, and it is necessary to ensure that the mobile beacons maintain a certain Line of Sight (LOS) with at least three fixed beacons at any point in space for localization. The mobile beacons are placed on the mobile robot and are used to obtain information about the position of the mobile robot. Mobile beacons and fixed beacons use the same hardware and are collectively referred to as localization beacons in this paper. For a location beacon, we can change its function in the driver to make it work as a mobile beacon or a fixed beacon, which is shown in Figure 5a. The location routing plays the role of a central controller in the MarvelMind positioning system. All the information measured by the beacons will be sent to the location routing, which will be processed and calculated by the location routing, as shown in Figure 5b.

**Figure 6:**
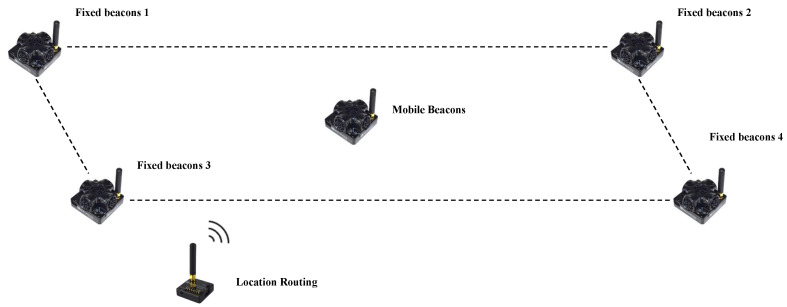
Example of MarvelMind 4 fixed beacon setting.

To ensure the accuracy of positioning, the built MarvelMind system should contain 3 to 4 fixed beacons. The system is built in the way shown in Figure 6. The fixed beacons can be mounted on walls or ceilings or fixed with brackets, and the maximum distance between fixed beacons is 50 m. The mobile beacons are placed on the object under test, and the mobile robot can transmit the positioning information acquired by the mobile beacons to the mobile robot’s host computer through the USB interface. The localization routing can be placed anywhere in the workspace and can work using a USB to power it.

**Figure 7:**
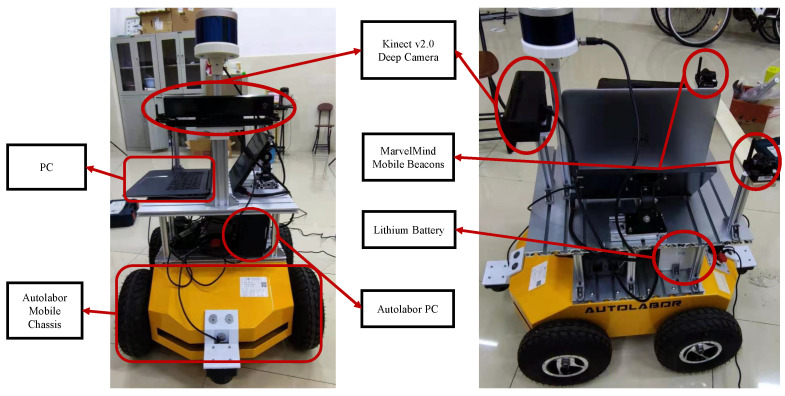
Mobile robot platform with experimental equipment.

Since the algorithm in this paper requires the placement of dual MarvelMind tags and also requires the mobile robot chassis to be equipped with a PC and connected with a network cable for distributed communication. Based on this need, the original AUTOLABOR mobile robot was modified to suit the experimental requirements of this paper. The modified mobile robot platform is shown in Figure 7, with the addition of dual MarvelMind location tags and an additional layer for the PC to ensure distributed information transmission using a network cable.

### 5.2. Experimental Design

In this section, we verify the effect of fused IPS back-end optimization algorithm by loop trajectory experiment and straight trajectory experiment. In the loop trajectory experiment, the results of loopback detection are used as the standard trajectory to compare the bit-pose trajectory results calculated by the back-end optimization algorithm of visual fusion IPS and ORB_SLAM2 back-end algorithm (without adding the back-end optimization of IPS) in the case of no loopback detection. And the straight trajectory experiment uses the straight line of the real scene as the standard trajectory to evaluate the accuracy of the back-end optimization algorithm of the fused IPS in this paper.

**Figure 8:**
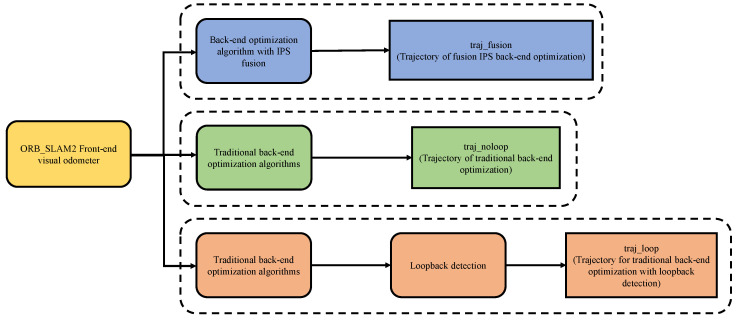
Experimental trajectory illustration diagram.

In the back-end global bit pose estimation experiment, in order to have the same initial value of odometer as the control group, the back-end optimization algorithm of fusion IPS uses the same VO part as the front-end to obtain the initial value of bit pose in this experiment as the control group. The experimental design is shown in Figure 8, where *traj_fusion* is the trajectory result obtained by the fused IPS back-end optimization algorithm proposed in this paper. *traj_fusion* trajectory is optimized without loopback detection. *traj_noloop* is the trajectory obtained directly by the traditional back-end optimization algorithm of ORB_SLAM2’s back-end optimization algorithm without fused IPS and without loopback detection. The *traj_loop* is the trajectory obtained by the traditional back-end optimization algorithm and optimized by loopback detection. *traj_loop* results have no cumulative drift and high accuracy due to the processing of loopback detection threads, which is used as the standard trajectory in the loop trajectory experiment.

### 5.3. Mobile Robot Platform Circular Trajectory Experiment

The experiment collected three groups of data with different travel trajectories. The travel distance of trajectory 1 is about 14 m, the travel distance of trajectory 2 is about 16.5 m, and the travel distance of trajectory 3 is about 17 m.

**Figure 9:**
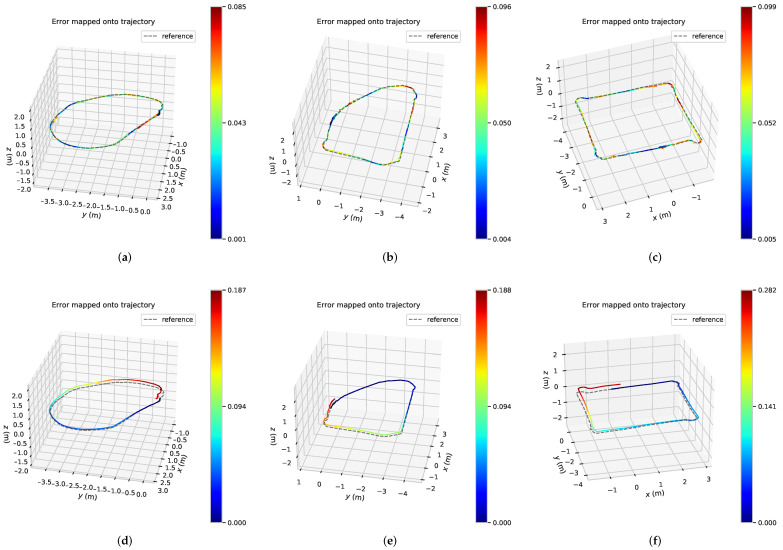
Circular trajectory error under different driving trajectories. (**a**) Our optimization algorithm (Trajectory 1); (**b**) Our optimization algorithm (Trajectory 2); (**c**) Our optimization algorithm (Trajectory 3); (**d**) ORB_SLAM2 back-end algorithm (Trajectory 1); (**e**) ORB_SLAM2 back-end algorithm (Trajectory 2); (**f**) ORB_SLAM2 back-end algorithm (Trajectory 3).

We used EVO-SLAM trajectory accuracy evaluation software [38] for visualization, and Figure 9 shows the error trajectories of the three sets of trajectory data collected, in which the dashed trajectories are the standard trajectories, i.e., *traj_loop* obtained by loopback detection optimization. Figure 9a–c shows the error trajectory obtained by the back-end optimization algorithm of visual fusion IPS, and Figure 9d–f shows the error trajectory obtained by the ORB_SLAM2 back-end algorithm. From the results of the three sets of trajectories, it can be seen that the trajectory *traj_noloop* obtained by the back-end optimization algorithm of the unfused IPS increases the error as the running time of the mobile robot increases, and the estimated poses are no longer in the same position when the robot returns to the starting position due to the cumulative error. In contrast, the trajectory *traj_fusion* obtained by the back-end optimization algorithm with IPS fusion has no cumulative drift in the overall trajectory due to the global positional constraints of IPS, and the starting and ending poses of the robot are also closer from the intuitive results of the trajectory.

**Figure 10:**
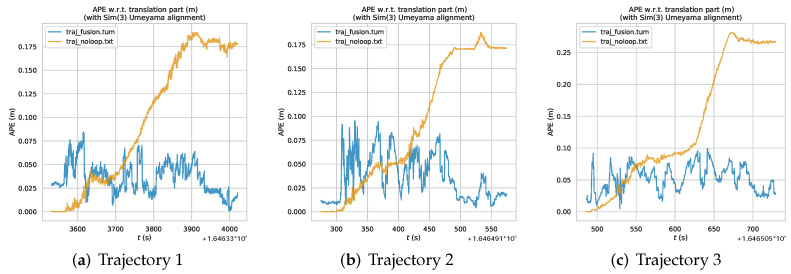
Absolute Pose Error of circular trajectory under different driving trajectories.

Figure 10 shows the trajectory error line plot of the loop trajectory plotting error with respect to time variation for different driving trajectories, and APE means Absolute Pose Error. It is obvious from the figure that the ORB_SLAM2 back-end algorithm results in increasing error with time change due to the cumulative error. In contrast, the trajectory error of the *traj_fusion* of the algorithm in this paper is more stable in a certain interval range of small floating, and does not appear constantly rising trend.

As can be seen from the trajectory error line graph, the back-end optimization algorithm incorporating IPS in this paper can effectively solve the cumulative drift problem of VSLAM and ensure the global consistency of estimated poses.

The maximum value (Max), standard deviation (Std), mean (Mean) and root-mean-squared error (RMSE) of the trajectory error are calculated for all three sets of data. The standard deviation can reflect the degree of deviation of the trajectory error from the mean, and the smaller the standard deviation, the less deviation from the mean. The root-mean-squared error needs to be calculated for all poses and the result is calculated by Equation (8):

(8) $$RMSE_{all}=\sqrt{\frac{1}{N}\sum_{i=1}^{N}{∥\log(T_{gt,i}^{-1}T_{est,i}{)}^{\vee }∥}_{2}^{2}}.$$

The error statistics of the three data sets in Table 1, Table 2 and Table 3. From the Max data, it can be seen that the error of the algorithm in this paper is optimized due to the exclusion of data affected by noise through the IPS outlier rejection algorithm, which ensures that the errors of the acquired positional trajectories are maintained below 0.1 m. In the data of Std *traj_fusion* value is smaller than *traj_noloop* value, which indicates that the floating degree of trajectory error of the back-end optimization algorithm of fusion IPS is smaller, and further indicates that the algorithm of this paper solves the problem of cumulative drift. From the Mean and RMSE data, it can be seen that the error between the back-end optimization algorithm of fusion IPS and the loopback detection result as the standard trajectory is only between 3 cm and 6 cm, so if the mobile robot performs a task in which the loopback trajectory cannot be driven, the back-end optimization algorithm of fusion IPS proposed in this paper can ensure that the trajectory accuracy obtained is close to the loopback detection optimization results, which in turn solves the cumulative drift problem of VSLAM under the loopbackless task.

### 5.4. Experiment on Straight Trajectory of Mobile Robot Platform

This experiment collects two sets of straight trajectories, the running speed of the two data carts are different, the speed of the mobile robot is 0.05 m/s, and 0.3 m/s respectively, and the distance of the carts travel is about 7 m. The acquired trajectory data, groundtruth indicates the trajectory true value, *traj_fusion* is the trajectory result obtained by the optimization algorithm in this paper. *traj_unloop* is the trajectory result obtained by ORB_SLAM2 back-end algorithm, the trajectory is not fused IPS nor loopback detection optimization.

**Figure 11:**
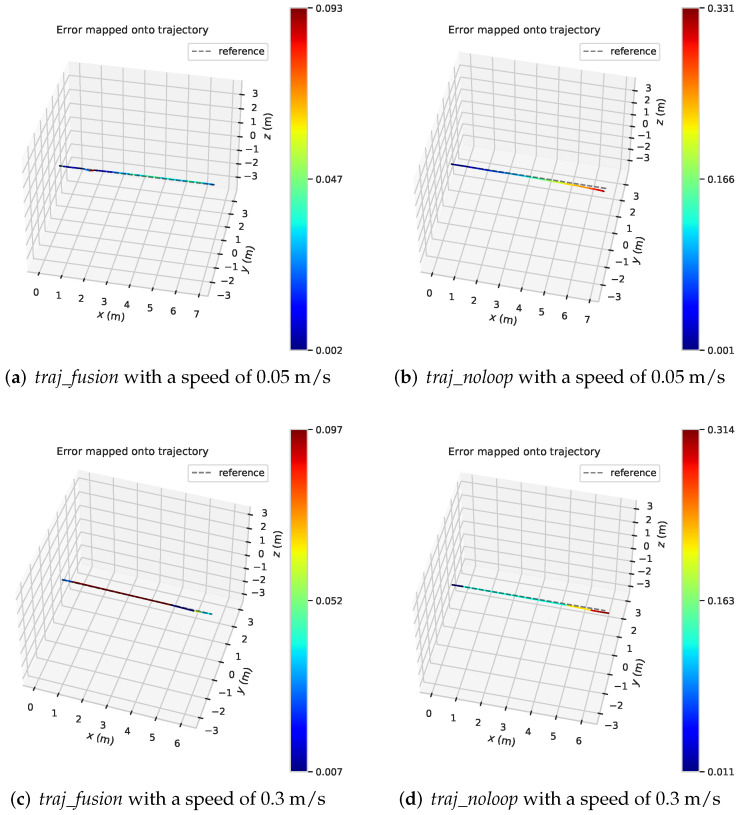
Trajectory error of straight trajectory at different speeds.

Calculating the trajectory error can get the results shown in Figure 11, which reflects the trajectory error of two sets of straight-line data, where the speed is 0.05 m/s in Figure 11a,b, and 0.3 m/s in Figure 11c,d. Figure 11a,c show the trajectory *traj_fusion* of fusion IPS, and Figure 11b,d shows the traditional back-end optimized trajectory *traj_noloop*. it can be seen in the figure that the *traj_noloop* trajectory also has the problem of cumulative drift in the straight line state, and the mobile robot runs in the *x*-axis and drifts in the *y*-axis. While *traj_fusion* trajectory results from the graph does not have a larger degree of positional drift.

**Figure 12:**
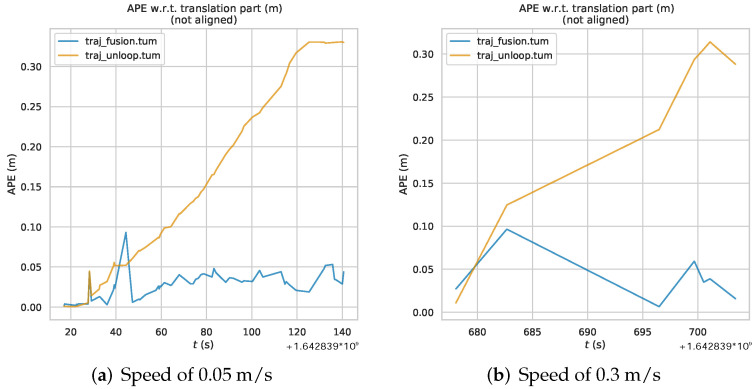
Absolute Pose Error of straight trajectory at different speeds.

From the results in the Figure 12, we can see that in the state of straight line driving, the error of *traj_fusion* trajectory of the algorithm after fusion IPS no longer increases with the growth of running time, and the overall error trend is relatively smooth, and the overall accuracy is maintained at about 0.05m, which effectively solves the problem of cumulative drift. And both sets of data show a similar trend, indicating that the running speed of the mobile robot does not have a large degree of influence on the accuracy of the positional trajectory of the fused IPS back-end optimization algorithm.

Similarly the maximum value (Max), Standard Deviation (Std), Mean (Mean) and Root-Mean-Squared Error (RMSE) are calculated for all data trajectory errors. The error statistics of the two sets of data in Table 4 and Table 5 can be obtained.

The maximum error of *traj_fusion* is also controlled below 0.1 m from the Max, which proves the effectiveness of IPS outlier suppression algorithm. And the *traj_fusion* Std is smaller than the result of *traj_noloop*, which indicates that the back-end optimization algorithm trajectory results of fusion IPS have smaller oscillation amplitude and no obvious cumulative drift phenomenon. From the results of Mean and RMSE, it can be seen that the error between *traj_fusion* trajectory and real trajectory can reach centimeter level, so the accuracy of *traj_fusion* can reach between 0.2 m and 0.4 m.

The analysis of the experimental results of the straight trajectory shows that the back-end optimization algorithm proposed in this paper can solve the cumulative drift problem of the VSLAM system, and the positioning accuracy of the algorithm can be maintained between 0.02 m and 0.03 m to meet the demand of indoor positioning. Moreover, since the algorithm can solve the cumulative drift problem of the VSLAM system without loopback detection, it can be used and maintain high accuracy in tasks where mobile robots cannot drive loopback trajectories.

## 6. Conclusions

SLAM is one of the key technologies for realizing research areas such as mobile robotics, autonomous driving, and mixed reality, and is also an important part of mobile robots to perceive their surroundings. This paper discusses the cumulative drift problem in VSLAM and the impact of the emergence of cumulative drift on VSLAM systems. A back-end optimization algorithm incorporating IPS is proposed for the many limitations of loopback detection methods for solving the cumulative error problem in the VSLAM framework. By comparing the experimental results of the circular trajectory and the straight trajectory of the mobile robot driving indoors, it can be seen that the back-end optimization algorithm of fusion IPS has a smaller error between the starting and ending positions and has a smaller error with the optimized trajectory of loopback detection, which can solve the cumulative drift problem instead of loopback detection. Therefore, the back-end optimization algorithm of the fused IPS proposed in this paper can solve the cumulative drift problem in the environment where loopback detection does not work, which extends the usage environment of the VSLAM system.

In this paper, the construction of the map is based on the purpose of indoor reconstruction only, and the method will also be limited by the sensor performance, and the accuracy will be affected, for example, the sensor range limits the size of the indoor environment, and the perception range of the depth camera likewise limits the scalability. We can only reach the upper limit of the sensors after fusion and cannot break through. Therefore, the next work will consider the optimization and improvement of the algorithm in this paper based on the productization application, so that the algorithm can automatically choose a more suitable algorithm according to the environment, and have the ability to switch the state automatically, such as when switching from indoor environment to outdoor environment, the algorithm can switch from IPS fusion to GPS. Meanwhile, we can build a 3D octree map by VSLAM, and build a better visual effect The dense point cloud map can better realize the localization and navigation of mobile robots.

## Figures and Tables

**Table 1:** Statistics of experimental results of trajectory 1.

Parameters (Unit: m)	Max	Std	Mean	RMSE
*traj_fusion*	0.0846	0.0164	0.0359	0.0395
*traj_noloop*	0.1900	0.0684	0.0946	0.1167

**Table 2:** Statistics of experimental results of trajectory 2.

Parameters (Unit: m)	Max	Std	Mean	RMSE
*traj_fusion*	0.0957	0.0230	0.0357	0.0424
*traj_noloop*	0.1880	0.0682	0.0932	0.1155

**Table 3:** Statistics of experimental results of trajectory 3.

Parameters (Unit: m)	Max	Std	Mean	RMSE
*traj_fusion*	0.0991	0.0203	0.0518	0.0556
*traj_noloop*	0.2816	0.1004	0.1384	0.1710

**Table 4:** Statistics of experimental results for speed of 0.05 m/s.

Parameters (Unit: m)	Max	Std	Mean	RMSE
*traj_fusion*	0.0929	0.0167	0.0280	0.0326
*traj_noloop*	0.3306	0.1127	0.1475	0.1856

**Table 5:** Statistics of experimental results for speed of 0.3 m/s.

Parameters (Unit: m)	Max	Std	Mean	RMSE
*traj_fusion*	0.0965	0.0279	0.0399	0.0487
*traj_noloop*	0.3141	0.1063	0.2213	0.2455

## Data Availability

Not applicable.
